# TGF-β induced reprogramming and drug resistance in triple-negative breast cells

**DOI:** 10.1186/s40360-022-00561-x

**Published:** 2022-04-08

**Authors:** Guoyu Wu, Yuchao Li

**Affiliations:** 1 Key Specialty of Clinical Pharmacy, The First Affiliated Hospital of Guangdong Pharmaceutical University, Guangzhou, China; 2 School of Clinical Pharmacy, Guangdong Pharmaceutical University, Guangzhou, China; 3 NMPA Key Laboratory for Technology Research and Evaluation of Pharmacovigilance, Guangdong Pharmaceutical University, Guangzhou, China; 4 MegaLab, MegaRobo Technologies Co., Ltd, Beijing, China

**Keywords:** Drug resistance, Triple-negative breast cancer, TGF-β, Transcriptional evolution

## Abstract

**Background:**

The development of drug resistance remains to be a major cause of therapeutic failure in breast cancer patients. How drug-sensitive cells first evade drug inhibition to proliferate remains to be fully investigated.

**Methods:**

Here we characterized the early transcriptional evolution in response to TGF-β in the human triple-negative breast cells through bioinformatical analysis using a published RNA-seq dataset, for which MCF10A cells were treated with 5 ng/ml TGF-β1 for 0 h, 24 h, 48 h and 72 h, and the RNA-seq were performed in biological duplicates. The protein-protein interaction networks of the differentially expressed genes were constructed. KEGG enrichment analysis, cis-regulatory sequence analysis and Kaplan-Meier analysis were also performed to analyze the cellular reprograming induced by TGF-β and its contribution to the survival probability decline of breast cancer patients.

**Result:**

Transcriptomic analysis revealed that cell growth was severely suppressed by TGF-β in the first 24 h but this anti-proliferate impact attenuated between 48 h and 72 h. The oncogenic actions of TGF-β happened within the same time frame with its anti-proliferative effects. In addition, sustained high expression of several drug resistance markers was observed after TGF-β treatment. We also identified 17 TGF-β induced genes that were highly correlated with the survival probability decline of breast cancer patients.

**Conclusion:**

Together, TGF-β plays an important role in tumorigenesis and the development of drug resistance, which implies potential therapeutic strategies targeting the early-stage TGF-β signaling activities.

**Supplementary Information:**

The online version contains supplementary material available at 10.1186/s40360-022-00561-x.

## Background

Cancer remains to be a leading cause of death worldwide. According to the estimation of IARC (The International Agency for Research on Cancer), there were 19.3 million new cases of cancer in 2020. Among them, female breast cancer was the most commonly diagnosed cancer, with about 2.3 million new cases [1]. TNBC (Triple-negative breast cancer) is an aggressive subtype of breast cancer, constituting 10–20% of all cases [2]. TNBC lacks all of the estrogen receptor (ER), progesterone receptor (PR) and HER2 receptor, thus is not eligible for anti-Her2 or hormone therapy. Chemotherapy remains to be the standard clinical treatment for TNBC patients and about 50% of them evolved drug resistance, leading to relapse [3].

Adaptive resistance, or acquired resistance, which arises in the course of therapy, could severely affect the prognosis of TNBC patients. Previous genomic studies reported that pre-existing resistance genotypes were adaptively selected by chemo drugs [4], while changes of transcriptional profile were acquired by reprogramming [5]. The non-genetic drug-tolerant state plays a critical role in the acquisition of drug resistance. One study showed that drug induced reprogramming was a complex multi-stage process, converting a transient transcriptional state to a stably resistant state [6]. The adaptation to drug usually happens within 3 days of treatment during which the drug-sensitive cells evade drug inhibition to proliferate [7]. The successfully evaded cancer cells normally go through a multi-stages process including cell cycle arrest, cellular reprogramming, drug resistance, immune cloaking, reactivation and metastatic relapse [8]. Especially, cell cycle arrest and subsequent cellular reprogramming are critical steps on the road to drug resistance and metastasis. A well-characterized cytokine that contributes to these two steps is TGF-β, which was found to play paradoxical roles in tumorigenesis: it act both as an anti-cancer agent that controls cell proliferation and as an oncogenic factor that promotes metastasis [9]. Recent studies imply that TGF-β induced quiescence contributes to the chemoresistance of tumors [10, 11]. However, previous studies mainly focused on long-term effects of TGF-β treatment [10, 12], the impact of TGF-β on the transcriptional profiles at early stages remains to be fully investigated. Is the seemingly opposing functions of TGF-β induced sequentially or at the same time? What are the key events contributing to the transition from a drug-sensitive state into a drug-resistance one?

To tackle these issues, we tried to unveil the early transcriptional evolution in response to TGF-β in a human triple-negative breast cell line through bioinformatical analysis on a RNA-seq dataset, for which MCF10A cells were treated with 5 ng/ml TGF-β1 for 0 h, 24 h, 48 h and 72 h, then the RNA-seq were performed in biological duplicates [13]. We first gained a global view on the transcriptomic dynamics of the cell line after TGF-β treatment. Transcriptomic analysis revealed a cellular reprogramming from a state of cell-cycle arrest to adaptation within the first 3 days of treatment. Interestingly, we found that the anti-proliferative and oncogenic effects of TGF-β happened at the same time frame. Sustained activation of several drug resistance markers was induced immediately after TGF-β stimulation. In addition, our results suggested that cellular reprograming induced by TGF-β contributes to the survival probability decline of breast cancer patients.

## Methods

### Data source

The RNA-seq dataset was obtained from NCBI’s Gene Expression Omnibus at https://www.ncbi.nlm.nih.gov/geo/query/acc.cgi?acc=GSE74377, reference number GSE74377 and the measurements were normalized by DESeq2 [13].

### Data analysis

The RNA-seq data matrices were processed in R (Version 4.0.3) [14]. We compared the treated MCF10A cells to the untreated cells as the reference, then differentially expressed genes were identified with the thresholds of | Fold change | > 1.5 and *p* value < 0.05. KEGG pathway enrichment analysis [15–17] of differentially expressed genes was performed using clusterProfiler [18] package in R(Version 4.0.3) [14]. The pathways with *p* value < 0.05 were considered significantly enriched.

The protein-protein interaction(PPI) networks of differentially expressed genes were built using the online tool STRING (https://string-db.org/) [19] and visualized using Cytoscape software (Version 3.8.2) [20]. The hub nodes of proteins were identified as proteins with degrees of connectivity over 50 in the PPI network. Cell compartment specific proteins encoded by differentially expressed genes were identified using Cytoscape software (Version 3.8.2) [21]. Cis-regulatory sequence was analyzed using Cytoscape plugin iRegulon [22]. Transcription factors were ranked in descending order based on the number of targets.

The Kaplan–Meier survival analysis was performed using the online tool Oncolnc (http://www.oncolnc.org/) [23]. For each gene, patients were assigned to 2 non-overlapping groups based on whether their gene expression levels were in the top 25% or bottom 25%.

## Results

### Characterization of the cellular transcriptome upon TGF-β treatment

**Fig. 1:**
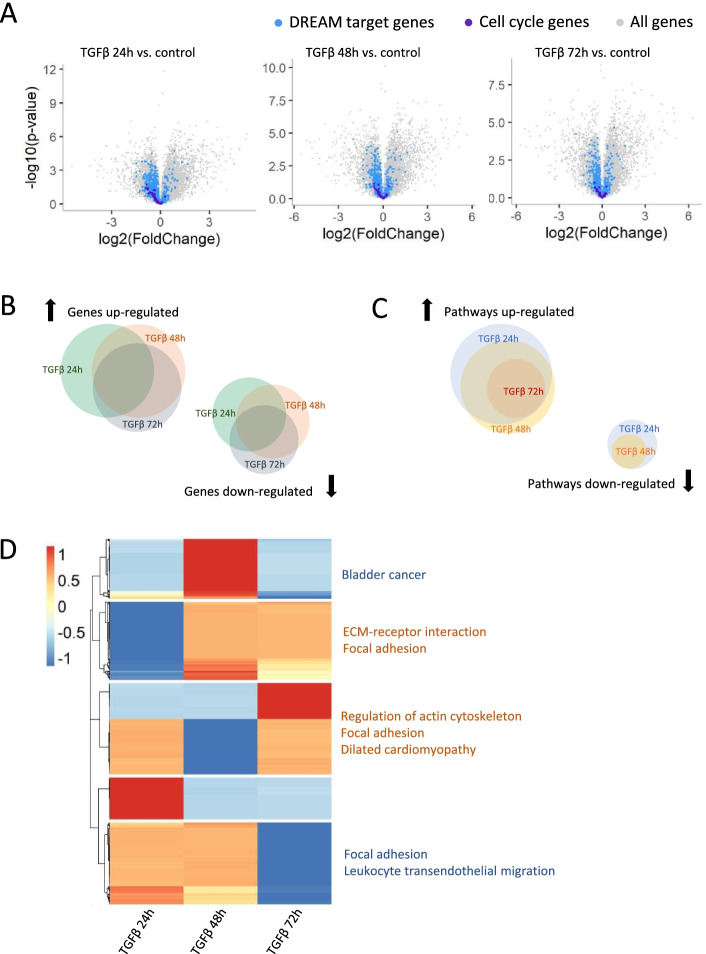
Transcriptome of TGF-β treated cells. **A** Volcano plot of the DREAM complex targets (blue) and cell cycle genes (purple). DREAM, Dimerization partner, RB-like, E2F4, and Multi-vulval class B. **B** Venn diagrams of gene sets that were differentially regulated. **C** Venn diagrams of pathways that were differentially regulated based on KEGG enrichment analysis [15–17]. Note: the area of each set does not strictly correlate with the number of genes contained within the set. **D** Clustered heatmap showing the dynamics of gene expression induced by TGF-β treatment. The values were centered and scaled in row direction. KEGG enrichment analysis was performed for each cluster [15–17]

We did bioinformatical analysis using a published RNA-seq dataset of MCF10A cells, which were treated with 5 ng/ml TGF-β1 for 0 h, 24 h, 48 h and 72 h [13]. The RNA-seq dataset was obtained from NCBI’s Gene Expression Omnibus (GSE74377). To evaluate the anti-proliferative effect of TGF-β, we analyzed the expression levels of 51 cell-cycle genes [7] and 971 DREAM (Dimerization partner, RB-like, E2F4, and Multi-vulval class B) targets, which includes transcriptional targets of E2F1/2/3 in the early cell cycle and targets of the MuvB related complexes such as MMB-FOXM1 in the late cell cycle [24, 25] (Supporting information, Tables S1 and S2). Twenty-four hours after TGF-β stimulation, the expressions of cell-cycle genes and DREAM targets were severely suppressed. However, this suppression attenuated throughout 48 h and 72 h (Fig. 1A), indicating the adaptation to the presence of TGF-β.

Next, we addressed the up-regulated and down-regulated genes after TGF-β treatment. Six hundred thirty-two genes were consistently down-regulated till 72 h but they were not enriched in any pathways (Fig. 1B and C). Nine hundred eighty-nine genes were consistently up-regulated till 72 h (Fig. 1B). The KEGG enrichment analysis [15–17] showed that 41, 32 and 23 pathways were significant up-regulated at 24 h, 48 h and 72 h, respectively(Supporting information, Tables S3 and S4). Notably, the pathways that were activated at 72 h, were consistently up-regulated throughout the first 3 days (Fig. 1C and Table 1). The up-regulation of pathways such as focal adhesion and ECM-receptor interaction, indicated that cells might interact with their extracellular environment more extensively after TGF-β stimulation (Table 1). Cell cycle arrest was induced in 24 h while up-regulated genes were consistently enriched in pathways in cancer throughout the first 72 h (Fig. 1A and C, Table 1). This observation suggested that TGF-β induces both anti-proliferative and oncogenic functions within 3 days.

**Table 1:** Pathways up-regulated at 72 h

Pathway ID	Pathway Name	Adjusted *p*-value
hsa04510	Focal adhesion	8.30E-10
hsa05200	Pathways in cancer	1.23E-05
hsa04512	ECM-receptor interaction	8.81E-05
hsa04360	Axon guidance	0.001244637
hsa00532	Glycosaminoglycan biosynthesis - chondroitin sulfate / dermatan sulfate	0.001386314
hsa04810	Regulation of actin cytoskeleton	0.001386314
hsa04540	Gap junction	0.001399944
hsa04380	Osteoclast differentiation	0.001515165
hsa00604	Glycosphingolipid biosynthesis - ganglio series	0.004333044
hsa05412	Arrhythmogenic right ventricular cardiomyopathy (ARVC)	0.004335863
hsa05222	Small cell lung cancer	0.005309891
hsa05414	Dilated cardiomyopathy	0.008779001
hsa05217	Basal cell carcinoma	0.008779001
hsa04916	Melanogenesis	0.008779001
hsa04520	Adherens junction	0.008779001
hsa05130	Pathogenic *Escherichia coli* infection	0.012094783
hsa05146	Amoebiasis	0.013140769
hsa05410	Hypertrophic cardiomyopathy (HCM)	0.024746656
hsa00520	Amino sugar and nucleotide sugar metabolism	0.03080917
hsa04670	Leukocyte transendothelial migration	0.031519599
hsa04010	MAPK signaling pathway	0.037721945
hsa04141	Protein processing in endoplasmic reticulum	0.048281202
hsa04144	Endocytosis	0.048281202

We then analyzed the dynamics of all the measured genes. The expression levels of each gene at 24 h, 48 and 72 h relative to the control samples were centered and scaled (Fig. 1D). Genes were then clustered according to their transcriptional profiles which was followed by KEGG enrichment analysis [15–17] for each cluster. The enrichment of focal adhesion and ECM-receptor interaction in multiple clusters is consistent with the above-mentioned findings.

### Protein-protein interaction network activation in response to TGF-β

**Fig. 2:**
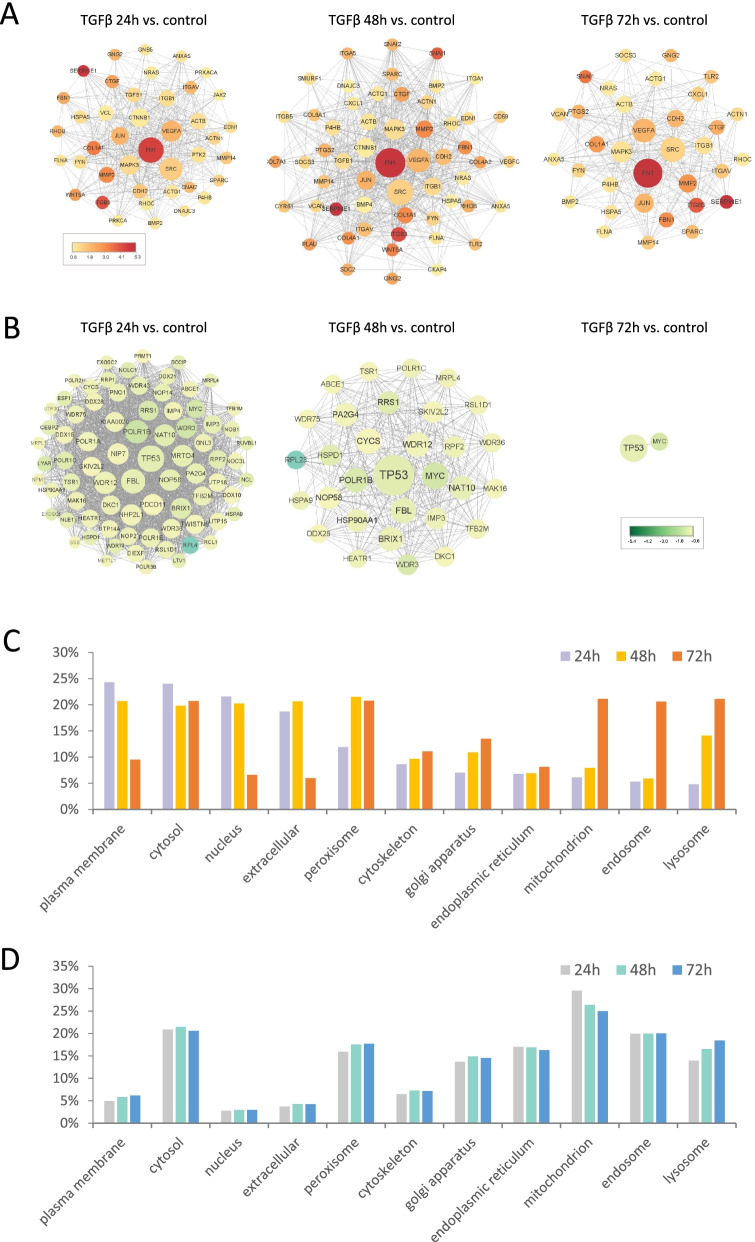
Protein-protein interaction network activation in response to TGF-β. **A** Hub nodes of up-regulated proteins in PPI networks. **B** Hub nodes of down-regulated proteins in PPI networks. The hub nodes were identified as proteins with degrees over 50. The degrees were represented by size of the circles. The values of Log2(FoldChange) of the genes correspond to the colors. PPI, Protein-protein interaction. **C** and **D** Cell compartment specific proteins encoded by differentially expressed genes across samples: **C** for up-regulated genes; **D** for down-regulated genes

Genes that differentially expressed after TGF-β treatment were used as inputs which was followed by PPI (Protein-protein interaction) networks construction using the online tool STRING [19]. The PPI networks of up-regulated proteins consisted of 1300, 1335, 1180 nodes and 8018, 8736, 6965 edges for 24 h, 48 h, 72 h, respectively (Fig. 2A); while the networks of down-regulated proteins consisted of 998, 962, 864 nodes and 7135, 4741, 2926 edges, respectively (Fig. 2B). Hub nodes with degrees over 50 were shown.

The results suggested that cells at 48 h were at a key stage with a giant PPI network for up-regulated proteins. Nodes with the highest degrees such as FN1, SRC, VEGFA, MAPK3 were suggested to be central to the PPI network throughout the time (Fig. 2A). FN1 encodes fibronectin, which is a glycoprotein involved in cell adhesion and migration [26, 27]. SRC is a proto-oncogene that regulates cell growth [27, 28]. VEGFA encodes a growth factor that could induce proliferation and migration [21, 27]. The protein encoded by MAPK3 is a member of the MAP kinase family, which acts in a signaling cascade that regulates cell cycle progression and cell differentiation [27, 29].

Consistent with the previous results that cells would gradually adapt to the presence of TGF-β, the number of down-regulated hub genes declined during the course of TGF-β treatment (Fig. 2B). Among the hub genes that present throughout the 3 days is TP53, which encodes a tumor suppressor protein and functions through inducing cell cycle arrest and DNA repair [27, 30].

To zoom in to the subcellular level of protein dynamics after TGF-β treatment, we categorized the differentially expressed genes according to their subcellular compartment and calculated the percentage of genes in each compartment [19]. The distributions of down-regulated proteins in specific compartments were consistent across the three samples (Fig. 2D). Interestingly, this is not the case for the up-regulated genes. During the period of 48 h after TGF-β treatment, the expression of proteins in extracellular, plasma membrane, cytosol and nucleus were more extensively induced, indicating enhanced cell-cell communication, signaling transduction and transcription in response to stimulus. After that, a large number of mitochondrial, endosome and lysosome proteins were highly expressed at 72 h (Fig. 2C). In addition to its primary role as an energy factory, mitochondria also plays a crucial role in cell signaling and cell growth regulation [31]. Endosome and lysosome proteins are important in cell proliferation [32]. These dynamics of proteins in each specific subcellular compartment supported the argument that cells started to adapt to the anti-proliferative effect of TGF-β in 48 h.

### TGF-β induced reprogramming contributes to drug resistance

**Fig. 3:**
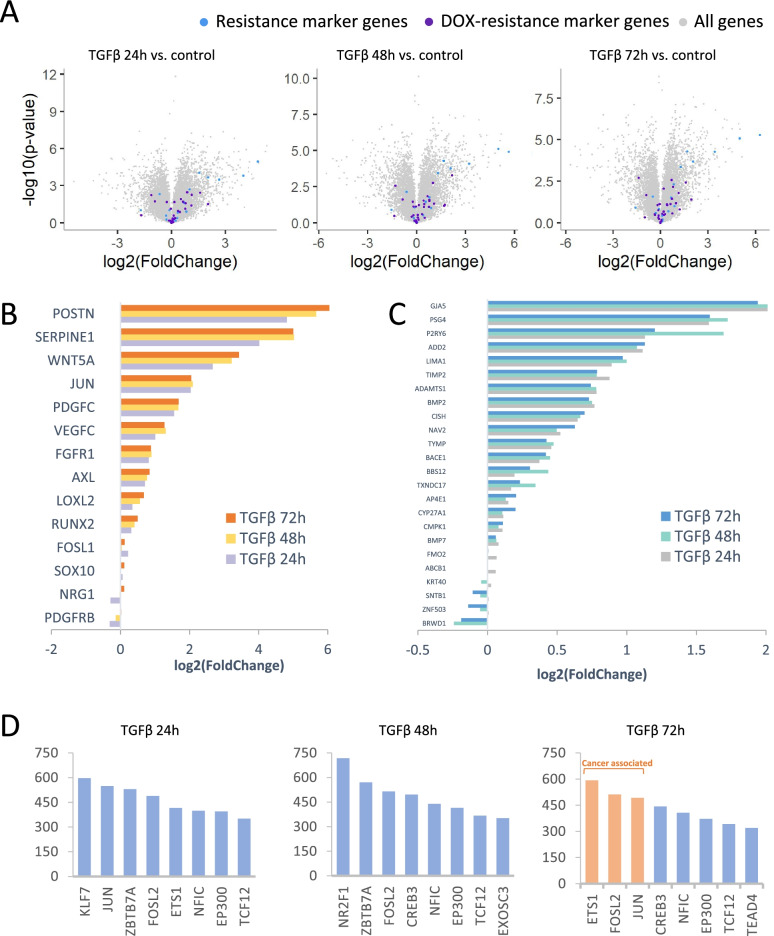
TGF-β induced reprogramming promotes drug resistance. **A** Volcano plot of resistance marker genes (blue) and DOX-resistance marker genes (purple). DOX, doxorubicin. Resistance marker genes: EGFR, NGFR, WNT5A, SERPINE1, POSTN, PDGFRB, NRG1, VEGFC, FOSL1, RUNX2, AXL, LOXL2, FGFR1, JUN, PDGFC, GAPDH, VGF, FGFR1, PDGFC, WNT5A, MITF, SOX10. DOX-resistance marker genes: ABCB1, AC011525.2, ADAMTS1, ADD2, ANGPT1, AP4E1, BACE1, BBS12, BMP2, BMP7, BRWD1, CISH, CMPK1, CRYBG2, CST1, CYP27A1, FAAH, FAT4, FMO2, FOXJ1, GJA5, HS3ST1, KRT40, LIMA1, MCPH1, NAV2, NSG2, P2RY6, PSG4, PTPRH, SLC38A2, SNTB1, STMN2, TIMP2, TRG-AS1, TXNDC17, TYMP, ZNF503. **B** Fold change in expression of resistance marker genes. **C** Fold change in expression of DOX-resistance marker genes. **D** Transcriptional factors of up-regulated genes based on cis-regulatory sequence analysis

To understand the role of TGF-β in the development of drug resistance, we analyzed the expression of well-known markers of drug resistance: EGFR, NGFR, WNT5A, SERPINE1, POSTN, PDGFRB, NRG1, VEGFC, FOSL1, RUNX2, AXL, LOXL2, FGFR1, JUN, PDGFC, GAPDH, VGF, FGFR1, PDGFC, WNT5A, MITF, SOX10 [6] (Fig. 3A). The results showed that 12, 12 and 14 resistance marker genes were highly expressed after 24 h, 48 h and 72 h TGF-β treatment, respectively (Fig. 3B). Chemotherapy is the standard treatment for many TNBC patients, and doxorubicin (DOX) is one of the widely used chemotherapeutic drugs [33]. Therefore, we here focused on the expression profiles of genes associated with resistance to doxorubicin: ABCB1, AC011525.2, ADAMTS1, ADD2, ANGPT1, AP4E1, BACE1, BBS12, BMP2, BMP7, BRWD1, CISH, CMPK1, CRYBG2, CST1, CYP27A1, FAAH, FAT4, FMO2, FOXJ1, GJA5, HS3ST1, KRT40, LIMA1, MCPH1, NAV2, NSG2, P2RY6, PSG4, PTPRH, SLC38A2, SNTB1, STMN2, TIMP2, TRG-AS1, TXNDC17, TYMP, ZNF503 [34] (Fig. 3A). Twenty-four, twenty, and twenty-one DOX-resistant markers were up-regulated after 24 h, 48 h and 72 h TGF-β treatment, respectively (Fig. 3C), which suggests that TGF-β treatment initiated the cellular transcriptional reprogramming of cells into a drug resistant state.

Next, we performed cis-regulatory sequence analysis for the up-regulated genes with iRegulon plugin [22] in Cytoscape [20]. Transcription factors with the largest number of targets were shown (Fig. 3D). Five hundred ninety-seven genes induced in the first 24 h were transcriptional targets of KLF7, which is reported as a tumor suppressor in breast cancer cells [35]. Seven hundred seventeen up-regulated genes at 48 h after TGF-β treatment were targets of NR2F1. NR2F1 has been demonstrated to contribute to cancer cell dormancy, as well as to be a potential impact on tumor recurrence and metastasis [36]. The master regulators of up-regulated genes at 72 h were regulators such as ETS1, FOSL2 and JUN, which have been reported as oncogenic transcription factors [37–39]. These results suggested a stepwise cellular reprogramming upon TGF-β stimulation.

### Cell reprograming induced by TGF-β has a negative impact on the survival probability of breast cancer patients

**Fig. 4:**
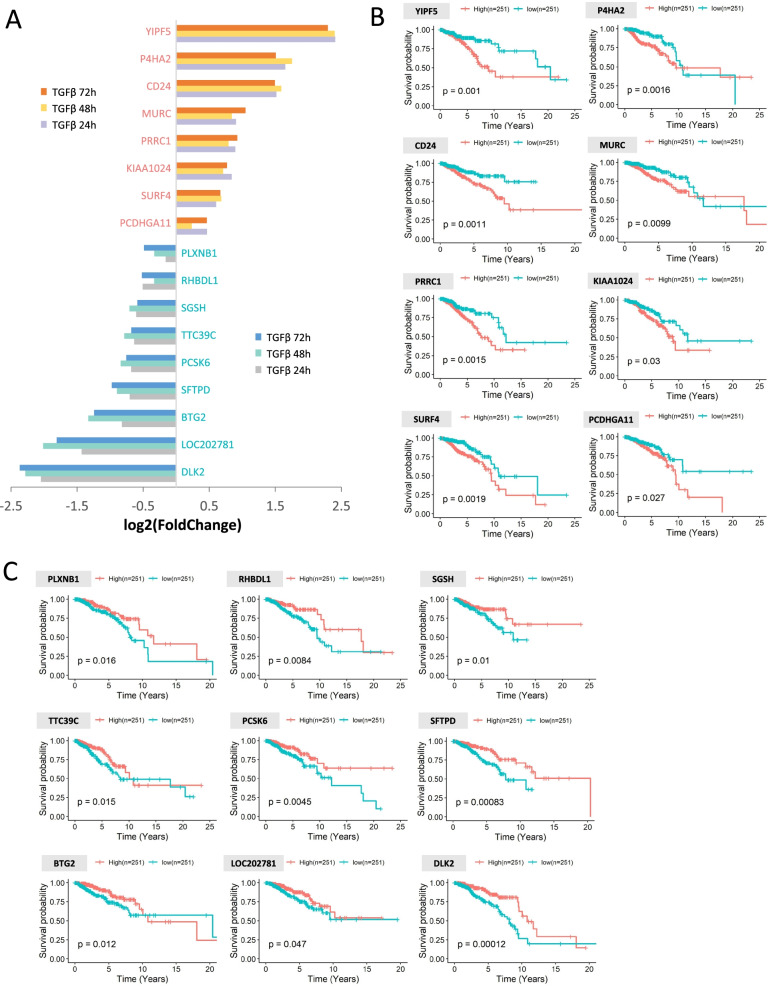
Transcription of genes associated with breast cancer upon TGF-β induction. **A** The expression level of genes correlated with survival probability of breast cancer patients. **B** Kaplan-Meier curves showed the overall survival was lower in patients with higher expression of those genes. **C** Kaplan-Meier curves showed the 5-year survival was lower in patients with lower expression of those genes. For each gene, patients were assigned to 2 non-overlapping groups based on whether their gene expressions were in the top 25% or bottom 25%

We further investigated the impact of TGF-β on the transcription of the genes associated with breast cancer. Kaplan-Meier analysis were performed using Oncolnc [23]. For each gene, patients were assigned to 2 non-overlapping groups based on whether the gene expression level was in the top 25% or bottom 25%. It turned out that 17 genes were highly correlated with the survival of breast cancer patients. Among them, YIPF5, P4HA2, CD24, MURC, PRRC1, KIAA1024, SURF4 and PCDHGA11 were up-regulated by TGF-β while negatively correlated with the survival (Fig. 4A and B). High level expressions of PLXNB1, RHBDL1, SGSH, TTC39C, PCSK6, SFTPD, BTG2, LOC202781 and DLK2, were significantly associated with increased survival probability, which were down-regulated by TGF-β (Fig. 4A and C).

## Discussion

Here, we provided comprehensive understandings of the early transcriptional evolution after TGF-β treatment in the human triple-negative breast cells. Cell growth was severely suppressed by TGF-β in the first 24 h but this anti-proliferate impact attenuated between 48 h and 72 h. The oncogenic actions of TGF-β happened within the same time frame with its anti-proliferative effects. We also identified 17 TGF-β induced genes that were highly correlated with the survival probability decline of breast cancer patients. In addition, sustained high expression of several drug resistance markers was observed after TGF-β treatment. Therefore, TGF-β plays an important role in tumorigenesis and the development of drug resistance.

Cells respond continually to their immediate environment by modulating gene expression. We observed that the total number of differentially expressed genes decreased in course of 72 h treatment. The expression of cell-cycle genes and DREAM targets were severely suppressed by TGF-β in the first 24 h, however, this anti-proliferate impact of TGF-β gradually diminished after 48 h. The attenuation could be explained by the refractory behavior of TGF-β signaling which is in turn determined by the receptor dynamics [40]. TGF-β signaling cascade initiates when the ligands bind to the TGF-β receptors. After that, Smads constantly shuttle between cytoplasm and nucleus, regulating the transcription of many genes [41]. A rapid depletion of receptors from the cell surface is triggered by ligand binding, which results in the internalization of both the receptors and ligands upon stimulation. The responsiveness to an acute TGF-β stimulus could be mitigated, for instance, in certain tumors with high autocrine signaling. The adaptation to the presence of TGF-β diminishes its anti-proliferate effect.

We found that the expression level of cell-cycle genes and DREAM targets were severely suppressed upon TGF-β stimulation. Cell cycle arrest were induced. As one of the reasonable explanations for the TGF-β induced drug-resistance, entry into quiescence triggered by TGF-β treatment could protect cells from damage by the drugs. Interestingly, we found that the cells in non-dividing state interacted with extracellular environment more extensively. The increased expression of complex plasma membrane-associated macromolecular assemblies and extracellular matrix receptors indicates an enhanced cell-cell communication after TGF-β treatment. Similar results were found in spontaneously quiescent cells, in which ECM components are upregulated, suggesting a more extensive interaction with their extracellular environment [24]. In addition, a number of genes associated with cell migration and cancer were persistently up-regulated. These observations are in agreement with the chemoresistant transcriptional programs previously identified in Triple-negative breast cancer patients [5]. The cellular reprogramming transforms the cells from a drug-sensitive state into a drug-resistant state.

The role of TGF-β in tumorigenesis has always been a controversial topic: friend or foe? TGF-β has long been considered as a paradoxical mediator of tumorigenesis for its paradoxical functions both as anti-proliferative and oncogenic regulators. Previous studies have shown that tumor cells with loss-of-function TGF-β signaling components would no longer be arrested by TGF-β [42]. Here, we found that TGF-β induced both the expression of cell growth inhibitory genes and cancer associated genes. However, the effects of cell cycle inhibition attenuate after 48 h while the oncogenic actions persistent throughout the 3 days. These results suggested that TGF-β contributes to the development of drug resistance via both of the two ways: inducing cell dormancy to protect cells from the damage of chemo drugs at an early stage, and initiating cellular reprogramming to evade the cell cycle blockage of the drugs. These carcinogenic events would happen within 3 days of TGF-β treatment and might seed development of permanent drug resistance. Reactive oxygen species (ROS) has been uncovered for its similar paradoxical tumorigenic role [43]: Low levels of ROS contribute to cell proliferation, differentiation and cell death [44], while high levels of ROS lead to oxidative stress which could cause genetic instability and may contribute to cancer initiation [45]. Interestingly, there is an interplay between TGF-β and ROS signaling throughout tumorigenesis and metastasis. Consistent with our results, TGF-β can induce ROS production in mitochondria and result in cell cycle arrest and apoptosis [46]. In turn, ROS stimulates the expression and secretion of TGF-β [43]. The anti-tumorigenic to pro-tumorigenic transition of ROS has been proved to be relevant in the context of TGF-β pathway activation. In cancer cells, TGFβ cross-talks extensively with ROS signaling which would enhance the invasive capacity [47]. Furthermore, the TGF-β - ROS interplay strongly contributes to cellular reprogramming and cancer initiation. Therapies targeting adaptive resistance would be extensively appreciated. New drug combinations targeting TGF-β cascade as well as its cross-talks, could be potential strategies to forestall future tumor relapse.

In addition to TGF-β, a number of factors and ligands are involved in drug resistance in triple-negative breast cancers, for instance, hepatocyte growth factor (HGF) could activate Met and induce metabolic reprogramming [48]. During the early stage of tumorigenesis, HGF is constitutively expressed to induce proliferation and angiogenesis. The engagement of HGF with c-MET activates signaling cascades related to invasion and epithelial to mesenchymal transition [49]. Intriguingly, TGF-β has been reported to regulate HGF-induced cell migration [50]. Negative interaction has been confirmed between TGF-β and HGF signaling pathways [51], which implies potential drug combination strategies targeting several interactive pathways in clinical application.

## Conclusions

Here, we provided a global view on the early transcriptional evolution of the human triple-negative breast cells after TGF-β treatment. The oncogenic actions of TGF-β happened within the same time frame with its anti-proliferative effects. Sustained activation of several drug resistance markers was induced immediately after TGF-β stimulation. Our studies suggest that TGF-β plays an important role in tumorigenesis and the development of drug resistance. New therapeutic strategies targeting the early-stage TGF-β signaling activities, could be a potential way to forestall drug resistance and tumor relapse.

## Supplementary Information

**Additional file 1: Table S1.** List of 51 cell-cycle genes. **Table S2.** List of DREAM targets. **Table S3.** List of pathways significantly up-regulated at 24 h after TGF-β treatment. **Table S4.** List of pathways significantly up-regulated at 48 h after TGF-β treatment.

## Data Availability

This study is a re-analysis of existing data that are publicly available from the NCBI’s Gene Expression Omnibus repository at https://www.ncbi.nlm.nih.gov/geo/query/acc.cgi?acc=GSE74377, reference number GSE74377.
