# Supplementary material for: TGF-β induced reprogramming and drug resistance in triple-negative breast cells
Source: BMC Pharmacol Toxicol. 2022 Apr 8;23:23. doi: 10.1186/s40360-022-00561-x (PMC8994282; doi:10.1186/s40360-022-00561-x)
Supplement: Supplementary file 1 — Additional file 1: Table S1. List of 51 cell-cycle genes. Table S2. List of DREAM targets. Table S3. List of pathways significantly up-regulated at 24 h after TGF-β treatment. Table S4. List of pathways significantly up-regulated at 48 h after TGF-β treatment. [file 40360_2022_561_MOESM1_ESM.docx]

Table S1. List of 51 cell-cycle genes

| ARHGEF39 | CCNE2 | E2F1 | MAD2L1 | RFC3 |
| --- | --- | --- | --- | --- |
| ATAD2 | CCNF | ESCO2 | MCM2 | RFC4 |
| AURKB | CDC20 | FAM83D | MCM4 | RRM2 |
| BIRC5 | CDC25A | GMNN | MCM5 | TACC3 |
| BRCA1 | CDC45 | GTSE1 | MCM6 | TOP2A |
| BUB1 | CDC6 | HJURP | MKI67 | TPX2 |
| BUB1B | CDK1 | HMMR | MYBL2 | UBE2C |
| CCNA2 | CDK2 | KIF11 | NUF2 |  |
| CCNB1 | CDKN3 | KIF20B | NUSAP1 |  |
| CCNB2 | CENPA | KIF23 | PLK1 |  |
| CCNE1 | CENPE | KIF4A | PRC1 |  |

Table S2. List of DREAM targets

| BUB1B | OIP5 | HIST1H2AH | ASF1B | NUP88 | NUP50 | SNRPD1 | GARS | EHBP1 | C2orf43 |
| --- | --- | --- | --- | --- | --- | --- | --- | --- | --- |
| CDCA3 | PIF1 | MSH5-SAPCD1 | CKAP5 | SUPT16H | CDK5RAP2 | TFAP4 | GPATCH4 | MYADM | C5orf30 |
| PLK1 | RAD51 | IFT80 | CDC7 | CUL3 | MYBL2 | POLE | HAUS7 | ANAPC1 | CIRH1A |
| TTK | CASC5 | RTTN | DSCC1 | DOLPP1 | SAPCD2 | C16orf59 | HMGN2 | DCAF15 | DCPS |
| PRC1 | CDK1 | SAE1 | TAF1A | GGCT | SSRP1 | SUV39H1 | HSPE1 | ILF3 | DDX46 |
| AURKA | NDC1 | SNRPA | ZNF367 | GINS4 | PASK | TFDP1 | IFRD2 | MAZ | DSCR3 |
| KIF4A | ESCO2 | HIST1H2BL | CEP295 | GTF3C2 | FAM161A | AGPAT5 | NRGN | NSMCE4A | EIF3B |
| NUF2 | EXOSC8 | CCDC34 | MTHFD1 | HIST1H2AI | KPNA2 | CBX5 | PBRM1 | PGP | EXOC4 |
| STIL | FANCD2 | CENPP | RHNO1 | HIST1H3C | RNF219 | NXT1 | PRKRIR | SENP1 | FKBP7 |
| CCNA2 | KNTC1 | FAM76B | BCL2L12 | HNRNPA1 | GART | SAAL1 | RAB8A | SIN3A | FOPNL |
| CCNB1 | ORC1 | RDM1 | GEN1 | KAT7 | TCOF1 | SMC1A | SMARCC1 | USP39 | G3BP1 |
| KIF11 | RBL1 | ZNF684 | MTBP | METTL2B | TMEM209 | NUP98 | STRA13 | WDR4 | GPATCH11 |
| NEK2 | TRAIP | BRD8 | NUP155 | MIS12 | ZMYM1 | CEP192 | TCEA1 | MARS | HACD2 |
| TPX2 | WDR62 | AC004381.6 | NUP85 | MSH3 | NUP160 | FAF1 | TONSL | MEIS2 | HADH |
| CDKN3 | ZGRF1 | MRE11A | HIST1H4C | PALB2 | HAUS3 | NUP62 | TOR1AIP1 | SLC1A5 | HIST1H1D |
| CENPF | CDC45 | SETD8 | ARL6IP1 | PIM1 | RCC1 | PBX3 | XRCC3 | CEP97 | HIST1H2BE |
| KIF18A | GSG2 | XRCC4 | C2orf69 | R3HDM1 | CASP2 | RFC1 | ZNF107 | EBP | HIST2H3A |
| RACGAP1 | MIS18BP1 | AFMID | LIN9 | SEC22C | SLC25A19 | CARHSP1 | ZNF92 | IQCB1 | HIST2H3D |
| CEP55 | PFAS | DLEU1 | ABCE1 | HIST1H2AM | MAGOHB | CCT5 | ZMYND19 | OXNAD1 | HSP90AA1 |
| FBXO5 | CDC6 | FOXRED1 | BAZ1B | LARP7 | RANBP1 | CEP57L1 | RNPS1 | RAD51B | KDM1A |
| BIRC5 | UBE2S | LOC642846 | SPATA5 | DCP2 | SUZ12 | CKLF | C5orf24 | SPATS2 | KLHDC4 |
| BUB1 | CKS1B | LSM3 | CKAP2 | HIST1H2BC | AMD1 | HAUS4 | HIST1H2BI | TMEM109 | LIAS |
| CCNB2 | CENPU | NFATC2IP | DHFR | KCTD9 | AK2 | LCORL | HNRNPH3 | ZNF695 | METTL16 |
| DEPDC1 | FEN1 | NUDT1 | NASP | OARD1 | USP28 | TTLL4 | HSPD1 | CDR2 | MRPL3 |
| GTSE1 | IQGAP3 | PPIH | RAD1 | HIST1H2AD | ODF2 | ZNF273 | KHDRBS1 | NUFIP2 | NF2 |
| KIF2C | MNS1 | SNRPB | ITGB3BP | TOP1 | RHEB | ATF7IP | ANKRD17 | ACD | PLSCR1 |
| ANP32E | CENPM | SRSF1 | PRIM2 | RAD51C | FANCL | CASP8AP2 | BYSL | EIF3A | PMF1 |
| ASPM | DDIAS | APOBEC3B | CACYBP | AEN | UACA | RECQL4 | COX20 | GTPBP2 | RFT1 |
| AURKB | RTKN2 | RAPGEF6 | CMC2 | E2F8 | ABHD2 | UBR7 | CWC27 | HIST1H2AB | RMND1 |
| CDC20 | ZWINT | DCLRE1C | ERI1 | CHAF1A | C9orf40 | CHRAC1 | CYP51A1 | HIST1H3J | SMARCA5 |
| CDC25A | GINS1 | FAM110A | NUP205 | MCM10 | FKBPL | GPR19 | DBP | HIST1H4L | TCTN2 |
| CDCA2 | SMC2 | HIST1H2AG | ORC3 | ARL6IP6 | GNL3 | HAUS1 | EXOSC3 | SHMT2 | THEM4 |
| DTL | TOP2A | DZIP3 | PRKDC | AUNIP | HNRNPH1 | HNRNPF | FAM178A | SMNDC1 | THOC1 |
| KIF14 | RNF26 | PCNA | SLC25A40 | POLA1 | L3MBTL2 | HNRNPR | GPR137C | THRAP3 | TMX1 |
| SGOL2 | CENPQ | KIF23 | ZNF473 | GINS2 | MRPL17 | LRRCC1 | KNOP1 | BCS1L | TNPO3 |
| ATAD2 | DBF4B | ANLN | NET1 | ARL13B | NAA15 | MYO19 | LAS1L | CEP85 | TUBG1 |
| KIF20A | FANCG | CENPA | RPA2 | MRTO4 | RBBP4 | NUP153 | LSM4 | EXOSC5 | TXNDC12 |
| LMNB1 | USP37 | MKI67 | KPNB1 | CDK2 | WDYHV1 | PKP4 | NUP54 | FAR1 | UTP15 |
| MAD2L1 | BRCA2 | MELK | MXD3 | FAM111B | ZNF146 | SMPD4 | NUPL1 | HSPA9 | VPRBP |
| NDC80 | INTS7 | TRIP13 | NUP43 | NOLC1 | HIST1H3H | TSEN15 | PYCRL | HSPB11 | ZNF724P |
| CENPL | MCM8 | UBE2C | CEP57 | POLR1E | MYC | HMGB3 | RRP9 | KHK | ETV5 |
| DLGAP5 | PSMC3IP | MCM2 | CEP78 | CBX3 | OPA1 | MDM1 | SMC5 | PAFAH1B3 | PKNOX1 |
| NCAPH | XRCC2 | MCM3 | HNRNPA0 | CENPJ | CENPC | FN3KRP | SNRNP25 | POLD2 | NOTCH2 |
| SGOL1 | C1orf112 | FOXM1 | RSRC1 | DNAJC9 | COQ7 | MYBL1 | SRRM1 | PTGES3 | EIF4E |
| TMPO | CCDC77 | MASTL | SP4 | POLE3 | LYRM7 | SLC29A1 | TTF1 | RAD23A | FNBP4 |
| MCM7 | CENPH | TIPIN | SFPQ | WRAP53 | CDKN2AIPNL | STAG1 | WDR46 | SLC1A4 | PNN |
| RFC3 | CENPK | CDCA5 | DDX10 | VRK1 | CNOT1 | TOP3A | WRN | TEX30 | NIPBL |
| SMC4 | CEP152 | ARHGEF39 | TBC1D31 | CMSS1 | KIF2A | CDKAL1 | ZNF267 | TMEM39B | AP2B1 |
| SPC25 | TK1 | MCM4 | WEE1 | NEDD1 | LONP1 | CENPW | RSBN1 | CCNE1 | BCAR3 |
| TICRR | PTTG1 | TCF19 | DNMT3B | SRSF7 | SF3B3 | CEP72 | PCF11 | LDLR | LCMT2 |
| HAUS6 | YEATS4 | DSN1 | HIST1H3D | TDP1 | TMEM18 | DDX39A | HIST1H1C | ZBTB14 | EFHC1 |
| CDC25C | DDX11 | NCAPD3 | HIST1H4A | HIRIP3 | XRCC6BP1 | DHX15 | HIST1H4H | ZNF207 | EAF2 |
| CDCA8 | HAT1 | NCAPG | TCERG1 | C1orf174 | ZNF45 | FIGN | KATNA1 | CNTROB | FBXL20 |
| ESPL1 | LSM5 | NCAPG2 | TMEM97 | HLTF | HN1 | HDGF | MAT2A | HIST1H4D | FRAT2 |
| HMMR | ATAD5 | RFC4 | ZRANB3 | LIN52 | RPL39L | HNRNPU | RPIA | CEP83 | IFNAR1 |
| ARHGAP11A | C5orf34 | SLBP | BRIX1 | TAF5 | TRIM45 | PTBP1 | SERTAD3 | DGCR8 | RNPC3 |
| CIT | EME1 | BARD1 | CCHCR1 | CEP135 | GABPB2 | TOMM40 | SSBP2 | GRPEL2 | ZNF518A |
| DEPDC1B | FAM111A | BRIP1 | CWF19L1 | HNRNPA2B1 | RBM8A | ZNF714 | TNPO2 | PRKAA1 | ARRDC3 |
| EXO1 | FANCM | DBF4 | DCAF16 | MED30 | DR1 | BCLAF1 | HSPA8 | SLC38A1 | PPP1R10 |
| FAM64A | KIF24 | NCAPD2 | GGH | HIST2H2AC | RBBP6 | HIST1H4E | JUND | SLFN11 | PPP3CB |
| CDCA7 | RQCD1 | RAD21 | HNRNPAB | REEP4 | HIST3H2A | ATF2 | SLC25A36 | TMEM138 | ZNF587 |
| CKAP2L | SASS6 | RMI1 | ING1 | CISD2 | RUNX1 | KIAA1715 | TOB2 | AIM1 | CDC42EP4 |
| NUSAP1 | E2F2 | USP1 | MIIP | PIGK | E2F7 | PHTF2 | ZNF24 | ALMS1 | IFIH1 |
| CHEK1 | KIAA0101 | DUT | POP7 | H2AFV | HIST2H2BE | AARS | PNRC2 | CAD | CFLAR |
| HJURP | METTL4 | C4orf46 | RMI2 | CREBZF | TACC3 | ATAD3A | SEC14L1 | CLIC4 | PMAIP1 |
| INCENP | ARHGAP11B | DCK | SFR1 | HIST1H2BH | SKA1 | EWSR1 | SS18 | EMG1 | GADD45A |
| PLK4 | CHEK2 | MIS18A | SMC6 | HIST1H2BM | ERCC6L | GATAD2A | RPS6KA5 | GPR180 | ANKRA2 |
| WDHD1 | GMNN | CDC25B | SRSF2 | GTPBP3 | MSH6 | HMGXB4 | USP53 | KPNA3 | TP53INP1 |
| CCDC150 | RRM1 | HMGB2 | UCHL5 | NUP93 | PHF19 | HS2ST1 | SMIM14 | NAP1L1 | EZH2 |
| SKA3 | DNA2 | UNG | CDKN2D | RAD9A | FANCC | IPO11 | STAT1 | NT5C3A | STK17B |
| CENPE | FANCB | KIF22 | AHCTF1 | SP1 | JADE1 | LRRC45 | BTG1 | NUDC | FAM72D |
| TROAP | ORC6 | DCLRE1A | HIST1H3G | GPD2 | ARHGAP19 | PIGW | WHSC1 | PATL1 |  |
| SHCBP1 | POC1A | KIF20B | ZNF551 | HIST1H2AK | MSH2 | PPP3R1 | PSRC1 | PGAP2 |  |
| SPAG5 | HELLS | POLE2 | CCDC18 | ACTL6A | CDT1 | PRIMPOL | KNSTRN | PPAN-P2RY11 |  |
| BRCA1 | HIST1H3B | FANCI | DDX21 | DDX20 | MPHOSPH9 | RNASEH2B | PRIM1 | RNF2 |  |
| FAM83D | HIST1H2AL | MND1 | HAUS5 | FUS | FIGNL1 | SLC16A1 | CDCA7L | RPL22L1 |  |
| PBK | MTF2 | RAD18 | HEATR1 | GRWD1 | SKP2 | SRSF10 | FANCA | SRSF3 |  |
| KIF15 | C14orf80 | RNASEH2A | KHSRP | HMBS | ECT2 | ZNF100 | FANCE | STIP1 |  |
| GINS3 | C17orf53 | TIMELESS | NCBP1 | ORC2 | LBR | FZR1 | HYLS1 | TBRG4 |  |
| PRR11 | C19orf40 | ANKRD32 | TRIM37 | XRCC1 | UHRF1 | SAP30 | PAICS | UBE2R2 |  |
| RAD51AP1 | C19orf48 | POLD1 | ZNF530 | AAAS | CDCA4 | HIST2H2AB | DPYSL2 | ZNF184 |  |
| CENPO | DARS2 | RIF1 | CCDC15 | ATAD3B | DEK | ASXL1 | EXOSC2 | ZNF85 |  |
| HAUS8 | HMGB1 | SPC24 | CSE1L | ATF4 | E2F1 | CCDC14 | HNRNPD | AHI1 |  |
| MCM5 | MTFR2 | STMN1 | HIST1H2AE | INIP | RFC2 | CTCF | PSIP1 | ABHD10 |  |
| H2AFX | MZT1 | CKS2 | APITD1 | MARCKS | PKMYT1 | DYRK1A | CDKN2C | ACACA |  |
| KIFC1 | PARPBP | CLSPN | CHCHD3 | RDX | TOPBP1 | FBXO4 | CCSAP | BIRC6 |  |
| RAD54L | POC5 | DCLRE1B | LRR1 | ZW10 | TRIM59 | HIST1H1B | GRK6 | EBAG9 |  |
| KIAA1524 | SKA2 | NIF3L1 | MUTYH | UBE2D3 | C18orf54 | HIST1H4I | KLHL23 | HCFC1 |  |
| BORA | TMEM194A | POLA2 | PEX3 | EIF2AK3 | DNMT1 | NAPEPLD | CLN6 | IFRD1 |  |
| RFC5 | TTI1 | POLQ | SLC3A2 | ZNFX1 | POLR3K | NCOA5 | FKBP5 | MRPS18B |  |
| SCLT1 | TTF2 | EXOSC9 | TCP1 | CCNF | SMCHD1 | SIVA1 | HAUS2 | PIGA |  |
| SPDL1 | HIST1H2BF | ZWILCH | BUB3 | PARP2 | SUV39H2 | TWISTNB | PDS5B | PTBP2 |  |
| UBE2T | HIST1H3F | CTDSPL2 | HIST1H2AC | GPSM2 | TRA2B | ZNF680 | PPAT | RAD23B |  |
| CENPN | NUCKS1 | H2AFZ | RANGAP1 | G2E3 | KIF18B | ARRB2 | PRPF4 | SPAG9 |  |
| DIAPH3 | NUDCD2 | LIN54 | POU2F1 | WDR76 | TUBB | AZIN1 | HNRNPDL | TARS |  |
| MDC1 | NOP58 | MMS22L | ALG10 | POLD3 | SYNCRIP | C12orf65 | NOC3L | ZNF138 |  |
| RRM2 | NUP107 | RFWD3 | HINT3 | GAS2L3 | GABPB1 | CAND1 | RASSF1 | ABCB10 |  |
| BLM | PCNT | SMC3 | HIST1H2AJ | MCM6 | GSTCD | CHTF18 | TUBA1C | ACP1 |  |
| NEIL3 | WBP11 | SNRNP40 | HIST1H2BO | RAD54B | NCAPH2 | DHTKD1 | TUBD1 | ARID2 |  |
| NUP35 | NPAT | TOE1 | HNRNPA3 | DTYMK | pk | ELP5 | CDK4 | BMPR1A |  |

Table S3. List of pathways significantly up-regulated at 24h after TGF-β treatment

| Pathway ID | Pathway Name | Ajusted p-value |
| --- | --- | --- |
| hsa04510 | Focal adhesion | 9.55E-14 |
| hsa04810 | Regulation of actin cytoskeleton | 2.18E-06 |
| hsa05200 | Pathways in cancer | 2.20E-06 |
| hsa04540 | Gap junction | 7.69E-06 |
| hsa04512 | ECM-receptor interaction | 0.000182937 |
| hsa04144 | Endocytosis | 0.000359541 |
| hsa04916 | Melanogenesis | 0.000512208 |
| hsa04360 | Axon guidance | 0.000739717 |
| hsa05412 | Arrhythmogenic right ventricular cardiomyopathy (ARVC) | 0.000811498 |
| hsa05414 | Dilated cardiomyopathy | 0.000811498 |
| hsa04380 | Osteoclast differentiation | 0.001257189 |
| hsa04520 | Adherens junction | 0.002114892 |
| hsa05130 | Pathogenic Escherichia coli infection | 0.002158647 |
| hsa04670 | Leukocyte transendothelial migration | 0.002253177 |
| hsa04010 | MAPK signaling pathway | 0.003114982 |
| hsa05217 | Basal cell carcinoma | 0.003901235 |
| hsa05100 | Bacterial invasion of epithelial cells | 0.003901235 |
| hsa05146 | Amoebiasis | 0.003996641 |
| hsa00532 | Glycosaminoglycan biosynthesis - chondroitin sulfate / dermatan sulfate | 0.00541962 |
| hsa05410 | Hypertrophic cardiomyopathy (HCM) | 0.005517359 |
| hsa04530 | Tight junction | 0.008430702 |
| hsa05215 | Prostate cancer | 0.010635154 |
| hsa04141 | Protein processing in endoplasmic reticulum | 0.012321218 |
| hsa05222 | Small cell lung cancer | 0.016902667 |
| hsa04370 | VEGF signaling pathway | 0.016902667 |
| hsa00604 | Glycosphingolipid biosynthesis - ganglio series | 0.019857958 |
| hsa04070 | Phosphatidylinositol signaling system | 0.019857958 |
| hsa05218 | Melanoma | 0.024342012 |
| hsa05110 | Vibrio cholerae infection | 0.025015045 |
| hsa00512 | Mucin type O-Glycan biosynthesis | 0.025015045 |
| hsa04310 | Wnt signaling pathway | 0.026670898 |
| hsa05214 | Glioma | 0.030514667 |
| hsa00520 | Amino sugar and nucleotide sugar metabolism | 0.031660291 |
| hsa00562 | Inositol phosphate metabolism | 0.03216123 |
| hsa04350 | TGF-beta signaling pathway | 0.032695526 |
| hsa04666 | Fc gamma R-mediated phagocytosis | 0.032943165 |
| hsa04012 | ErbB signaling pathway | 0.03766931 |
| hsa05219 | Bladder cancer | 0.03925361 |
| hsa05211 | Renal cell carcinoma | 0.044045859 |
| hsa05213 | Endometrial cancer | 0.044608767 |
| hsa04912 | GnRH signaling pathway | 0.049305523 |

Table S4. List of pathways significantly up-regulated at 48h after TGF-β treatment

| Pathway ID | Pathway Name | Ajusted p-value |
| --- | --- | --- |
| hsa04510 | Focal adhesion | 7.31E-13 |
| hsa05200 | Pathways in cancer | 1.37E-08 |
| hsa04810 | Regulation of actin cytoskeleton | 5.57E-06 |
| hsa04512 | ECM-receptor interaction | 6.09E-06 |
| hsa05412 | Arrhythmogenic right ventricular cardiomyopathy (ARVC) | 0.000176683 |
| hsa04540 | Gap junction | 0.000176683 |
| hsa05217 | Basal cell carcinoma | 0.000176683 |
| hsa04360 | Axon guidance | 0.000536874 |
| hsa04916 | Melanogenesis | 0.00080845 |
| hsa04520 | Adherens junction | 0.001202063 |
| hsa05414 | Dilated cardiomyopathy | 0.001398296 |
| hsa00532 | Glycosaminoglycan biosynthesis - chondroitin sulfate / dermatan sulfate | 0.001658971 |
| hsa05410 | Hypertrophic cardiomyopathy (HCM) | 0.004538796 |
| hsa04380 | Osteoclast differentiation | 0.005252558 |
| hsa04144 | Endocytosis | 0.005252558 |
| hsa05146 | Amoebiasis | 0.007828733 |
| hsa05215 | Prostate cancer | 0.007828733 |
| hsa04670 | Leukocyte transendothelial migration | 0.008899199 |
| hsa05130 | Pathogenic Escherichia coli infection | 0.008899199 |
| hsa05213 | Endometrial cancer | 0.01121921 |
| hsa05222 | Small cell lung cancer | 0.01121921 |
| hsa04142 | Lysosome | 0.011234404 |
| hsa04010 | MAPK signaling pathway | 0.019667551 |
| hsa04141 | Protein processing in endoplasmic reticulum | 0.022220478 |
| hsa00604 | Glycosphingolipid biosynthesis - ganglio series | 0.025308824 |
| hsa04350 | TGF-beta signaling pathway | 0.025308824 |
| hsa04530 | Tight junction | 0.027698625 |
| hsa00512 | Mucin type O-Glycan biosynthesis | 0.034413403 |
| hsa05100 | Bacterial invasion of epithelial cells | 0.034413403 |
| hsa05110 | Vibrio cholerae infection | 0.034413403 |
| hsa00520 | Amino sugar and nucleotide sugar metabolism | 0.046008795 |
| hsa00562 | Inositol phosphate metabolism | 0.047687574 |
